# A New High-Order Stable Numerical Method for Matrix Inversion

**DOI:** 10.1155/2014/830564

**Published:** 2014-02-06

**Authors:** F. Khaksar Haghani, F. Soleymani

**Affiliations:** ^1^Department of Mathematics, Shahrekord Branch, Islamic Azad University, Shahrekord, Iran; ^2^Department of Mathematics, Zahedan Branch, Islamic Azad University, Zahedan, Iran

## Abstract

A stable numerical method is proposed for matrix inversion. The new method is accompanied by theoretical proof to illustrate twelfth-order convergence. A discussion of how to achieve the convergence using an appropriate initial value is presented. The application of the new scheme for finding Moore-Penrose inverse will also be pointed out analytically. The efficiency of the contributed iterative method is clarified on solving some numerical examples.

## 1. Introduction and Preliminary Notes

It is well known that the inverse of a square matrix *A*_*m*×*m*_, which is also known as a reciprocal matrix, is a matrix *A* ^−1^ such that *AA* ^−1^ = *I*, where *I* is the identity matrix. A regular nonsingular matrix *A*_*m*×*m*_ can be inverted using methods such as the Gauss-Jordan elimination or Gaussian elimination method. Such schemes fall in the category of direct methods for this purpose.

The direct methods cannot properly handle sparse matrices possessing sparse inverses arising in the numerical solution of integral equations [1]. On the other hand, methods such as conjugate gradient for symmetric positive definite matrices and GMRES are effective for large sparse linear systems. However, there is a problem when the coefficient matrix (when solving a linear system of equations) is ill-conditioned. To remedy this, one may apply a preconditioner to the system in which its construction is not an easy task [2].

An iterative method for preconditioning is the SPAI (sparse approximate inverse preconditioner) algorithm [3]. Given a sparse matrix *A* the SPAI algorithm computes a sparse approximate inverse *M* by minimizing ||*AM* − *I*|| in the Frobenius norm. Then, the approximate inverse is computed explicitly and can be applied as a preconditioner to an iterative method.

There are other types of schemes, which can be considered as iteration methods while they have different structures; see, for example, [4, 5]. In such iterative methods, at each iteration an approximate inverse of a matrix (if it is rectangular, one can find Moore-Penrose inverse) may easily be attained. And consequently, the users have the ability to solve the linear systems (with multiple right-hand side vectors) iteratively or use the approximate inverses in sensitivity analysis and the preconditioning of a linear system. This type of methods is in focus here.

Several known methods were proposed for approximating matrix inverse, such as those based on the so-called minimum residual iterations and Hotelling-Bodewig algorithm [6]. The Hotelling-Bodewig algorithm is defined by

(1) $$\begin{array}{c} V_{n+1}=V_{n}\left(2I-AV_{n}\right),\;n=0,1,2,\ldots , \end{array}$$

where *I* is the identity matrix. Schulz in [7] found that the eigenvalues of *I* − *AV*_0_ must have magnitudes less than 1 to ensure the convergence, which is a key element in designing higher efficient Schulz-type iterative methods.

In 2011, Li et al. in [8] theoretically investigated

(2) $$\begin{array}{c} V_{n+1}=V_{n}\left(3I-AV_{n}\left(3I-AV_{n}\right)\right),\;n=0,1,2,\ldots , \end{array}$$

and also proposed another third-order iterative method for finding *A* ^−1^ as follows:

(3) $$\begin{array}{c} V_{n+1}=\left[I+\frac{1}{4}\left(I-V_{n}A\right){\left(3I-V_{n}A\right)}^{2}\right]V_{n},\;n=0,1,2,\ldots . \end{array}$$

The iterative method (2) can also be found in Chapter 5 of the textbook [9]. As an another example from this primary source, the authors provided the following twelfth-order method:

(4) Vn+1=Vn(I+Yn(I+Yn(I+Yn(I+Yn(I+Yn(I+Yn(I+Yn(I+Yn(I+Yn(I+Yn(I+Yn))))))))))), n=0,1,2,…,

in which *Y*_*n*_ = *I* − *AV*_*n*_. For further reading, one may refer to [10–12].

In this paper, we will propose an efficient iterative method for finding *A* ^−1^ numerically. The theoretical convergence of the method will also be studied. We also discuss the application of the new scheme in finding Moore-Penrose inverse (also known as pseudoinverse) for rectangular or singular matrices. It is also proven analytically that the new method has asymptotical stability in general. Some large-scale sparse matrices will be taken into account as some examples to put on show a clear reduction of the execution time when the new algorithm is applied.

The rest of the paper is organized as follows. The main contribution of this paper is given in Sections 2-3.  Section 2 is devoted to the analysis of convergence which shows that the method can be considered for the pseudoinverse as well.  Section 3 thoroughly and for the first time studies the stability of this high-order Schulz-type iterative method for finding generalized inverses.  Section 4 covers the matter of initial guess/value in order to preserve the convergence order. Subsequently, the method is examined in Section 5 numerically. And finally, concluding remarks are presented in Section 6.

## 2. A New Method and Its Convergence Study

By applying the following nonlinear equation solver (to see the new developments on root-finding methods, refer to [13])

(5) yn=xn−2−1f′(xn)−1f(xn),zn=xn−f(yn)−1f(xn),un=zn−[[zn−xn]−1(f(zn)−f(xn))]−1f(zn),gn=un−f(un)−1f(un),xn+1=gn−[[gn−un]−1(f(gn)−f(un))]−1f(gn), n=0,1,2,…,

on the nonlinear matrix equation *AV* = *I*, we obtain a fixed-point iteration for matrix inversion using *ψ*_*n*_ = *AV*_*n*_ as follows:

(6) Vn+1=164Vnϕ(Vn)=164Vn(816I−4812ψn+17393ψn2−43044ψn3 +77154ψn4−103312ψn5+105039ψn6 −81576ψn7+48268ψn8−21516ψn9 +7071ψn10−1652ψn11+258ψn12 −24ψn13+ψn14).

Simplifying (6) by proper factorizing yields

(7) $$\begin{array}{c} \zeta_{n}=17I+\psi_{n}\left(-28I+\psi_{n}\left(22I+\psi_{n}\left(-8I+\psi_{n}\right)\right)\right),\\ \kappa_{n}=\psi_{n}\zeta_{n},\\ V_{n+1}=\frac{1}{64}V_{n}\zeta_{n}\left(48I+\kappa_{n}\left(-12I+\kappa_{n}\right)\right),\;n=0,1,2,\ldots , \end{array}$$

wherein the sequence of iterates {*V*_*n*_}_*n*=0_^*n*=*∞*^ converges to *A* ^−1^ for a good initial value. Such a guess, *V*_0_, will be discussed in Section 4.

**Theorem 1**

Let *A* = [*a*_*ij*_]_*m*×*m*_ be a nonsingular real or complex matrix. If the initial approximation *V*_0_ satisfies

(8) $$\begin{array}{c} ||I-AV_{0}||<1, \end{array}$$

then the iterative method (7) converges with at least twelfth order to *A* ^−1^.

**Proof**

Let ||*I* − *AV*_0_|| < 1, and for the sake of simplicity assume that *E*_0_ = *I* − *AV*_0_ and *E*_*n*_ = *I* − *AV*_*n*_ = *I* − *ψ*_*n*_ stand for the symmetric residual matrix. It is straightforward to have

(9) En+1=I−AVn+1=I−A(164Vn(816I−4812ψn+17393ψn2−43044ψn3 +77154ψn4−103312ψn5+105039ψn6 −81576ψn7+48268ψn8−21516ψn9 +7071ψn10−1652ψn11 +258ψn12−24ψn13+ψn14))=−164(−4I+ψn)3(−I+ψn)12=164(3I+I−ψn)3(I−ψn)12=164(3I+En)3En12=164(27En12+27En13+9En14+En15).

Hence, we attain

(10) $$\begin{array}{c} ||E_{n+1}||\leq \frac{1}{64}\left(27{||E_{n}||}^{12}+27{||E_{n}||}^{13}+9{||E_{n}||}^{14}+{||E_{n}||}^{15}\right). \end{array}$$

In addition, since ||*E*_0_|| < 1, by relation (10) and using mathematical induction, we obtain that ||*E*_1_|| ≤ (1/64)(27||*E*_0_||^12^ + 27||*E*_0_||^13^ + 9||*E*_0_||^14^ + ||*E*_0_||^15^) ≤ ||*E*_0_||^12^ < 1. If we consider ||*E*_*n*_|| < 1, therefore

(11) ||En+1||≤164(27||En||12+27||En||13+9||En||14+||En||15)≤||En||12.

Furthermore, we get that

(12) $$\begin{array}{c} ||E_{n+1}||\leq {||E_{n}||}^{12}\leq \cdots \leq {||E_{0}||}^{12^{n+1}}<1. \end{array}$$

That is, *I* − *AV*_*n*_ → 0, when *n* → *∞*, and thus *V*_*n*_ → *A* ^−1^, as *n* → *∞*.

Now, we must show the twelfth order using the sequence {*V*_*n*_}_*n*=0_^*n*=*∞*^. To do this, we denote *ε*_*n*_ = *V*_*n*_ − *A* ^−1^ as the error matrix in the iterative procedure (7). We have

(13) I−AVn+1=164[27(I−AVn)12+27(I−AVn)13  +9(I−AVn)14+(I−AVn)15].

Hence, we could get that

(14) A(A−1−Vn+1) =164[27A12(A−1−Vn)12+27A13(A−1−Vn)13     +9A14(A−1−Vn)14+A15(A−1−Vn)15].

By multiplying *A* ^−1^ by the left side, we have

(15) A−1−Vn+1 =164[27A11(A−1−Vn)12+27A12(A−1−Vn)13     +9A13(A−1−Vn)14+A14(A−1−Vn)15],

which now by taking an arbitrary matrix norm results in

(16) ||εn+1||≤164[27||A||11||εn||12+27||A||12||εn||13    +9||A||13||εn||14+||A||14||εn||15].

And thus

(17) ||εn+1||=(164[27||A||11+27||A||12||εn||1  +9||A||13||εn||2+||A||14||εn||3])||εn||12.

That is, the iteration (7) converges with at least twelfth order to *A* ^−1^. This concludes the proof.

At this time, we discuss an application of (7) for finding the generalized inverses. The Moore-Penrose inverse of a complex matrix *A* ∈ *ℂ* ^*m*×*k*^ (also called pseudoinverse), denoted by *A* ^†^ ∈ *ℂ* ^*k*×*m*^, is a unique matrix *V* ∈ *ℂ* ^*k*×*m*^ satisfying the following four Penrose equations:

(18) $$\begin{array}{c} AVA=A,\;\;VAV=V,\\ {\left(AV\right)}^{*}=AV,\;\;{\left(VA\right)}^{*}=VA, \end{array}$$

wherein A* is the conjugate transpose of *A*. Ben-Israel and his colleagues in [14, 15] used the method (1) with the starting value

(19) $$\begin{array}{c} V_{0}:=\alpha A^{*}, \end{array}$$

where 0 < *α* < 2/*ρ* (A*A) and *ρ* (·) denotes the spectral radius. The authors in [15] further investigated that the sequence generated by

(20) $$\begin{array}{c} V_{n}=\alpha \overset{n}{\underset{i=0}{\sum }}A^{*}{\left(I-\alpha AA^{*}\right)}^{i},\;n=0,1,2,\ldots , \end{array}$$

converges to the pseudoinverse.

In the following theorem, we show analytically that in case of having singular or rectangular matrices, scheme (7) by considering the initial approximation (19) converges to the Moore-Penrose generalized inverse.

**Theorem 2**

For the sequence {*V*_*n*_}_*n*=0_^*n*=*∞*^ generated by the iterative Schulz-type method (7), and any *n* ≥ 0, it holds that

(21) $$\begin{array}{c} {\left(AV_{n}\right)}^{*}=AV_{n},\;\;{\left(V_{n}A\right)}^{*}=V_{n}A,\\ V_{n}AA^{†}=V_{n},\;\;A^{†}AV_{n}=V_{n}. \end{array}$$

**Proof**

We will prove the conclusion by induction on *n*. For *n* = 0, and considering (19), the first two equations of (21) can be demonstrated simply. And thus, we only give a verification to the last two equations as follows:

(22) V0AA†=αA∗AA†=αA∗(AA†)∗=αA∗(A†)∗A∗=α(AA†A)∗=αA∗=V0,A†AV0=(A†A)αA∗=α(A†A)∗A∗=αA∗(A†)∗A∗=α(AA†A)∗=αA∗=V0.

Assume now that the conclusion holds for some *n* > 0. We now show that it continues to hold for *n* + 1. Using the iterative method (7), one has

(23) (AVn+1)∗ =[A(164Vnϕ(Vn))]∗ =164(816ψn∗−4812(ψn∗)2+17393(ψn∗)3 −43044(ψn∗)4+77154(ψn∗)5−103312(ψn∗)6 +105039(ψn∗)7−81576(ψn∗)8+48268(ψn∗)9 −21516(ψn∗)10+7071(ψn∗)11−1652(ψn∗)12 +258(ψn∗)13−24(ψn∗)14+(ψn∗)15) =164[816ψn−4812ψn2+17393ψn3−43044ψn4 +77154ψn5−103312ψn6+105039ψn7 −81576ψn8+48268ψn9−21516ψn10 +7071ψn11−1652ψn12+258ψn13−24ψn14+ψn15] =A(164Vnϕ(Vn)) =AVn+1,

where the following fact (*AV*_*n*_)* = *AV*_*n*_ has been used. Thus, the first equality in (21) holds for *n* + 1, and the second equality can be proved in a similar way. For the third equality in (21), using the assumption that *V*_*n*_ *AA* ^†^ = *V*_*n*_ and the iterative method (7), we could write down

(24) Vn+1AA† =[164Vnϕ(Vn)]AA† =164(816VnAA†−4812VnψnAA†+17393Vnψn2AA† −43044Vnψn3AA†+77154Vnψn4AA† −103312Vnψn5AA†+105039Vnψn6AA† −81576Vnψn7AA†+48268Vnψn8AA† −21516Vnψn9AA†+7071Vnψn10AA† −1652Vnψn11AA†+258Vnψn12AA† −24Vnψn13AA†+Vnψn14AA†) =164(816Vn−4812Vnψn+17393Vnψn2 −43044Vnψn3+77154Vnψn4−103312Vnψn5 +105039Vnψn6−81576Vnψn7+48268Vnψn8 −21516Vnψn9+7071Vnψn10−1652Vnψn11 +258Vnψn12−24Vnψn13+Vnψn14) =164Vnϕ(Vn) =Vn+1.

Hence, the third equality in (21) holds for *n* + 1. The fourth equality can similarly be proved, and the desired result follows.

The iterative method (7) is a matrix multiplication rich scheme. So, in order to reduce the computational load of matrix multiplications, it is enough to use SparseArray[mat] to avoid unnecessary multiplications to the nonzero elements when dealing with large sparse matrices.

**Remark 3**

The (inverse finder) informational efficiency index is defined as *p*/*η* where *p* and *η* stand for the local order and the number of matrix-matrix products per cycle. The proposed method of this paper requires 8 matrix-matrix multiplications to achieve the convergence order 12. This implies that 12/8 = 1.5 as its informational index, which is much better than 2/2 = 1, 3/3 = 1, 3/4 = 0.75, and 12/12 = 1 of schemes (1), (2), (3), and (4), respectively.

Before stating the main theorem for finding Moore-Penrose inverse, it is required to recall that for *A* ∈ *ℂ* ^*m*×*k*^ with the singular values *σ*_1_ > *σ*_2_ > ⋯*σ*_*r*_ > 0 and the initial approximation *V*_0_ = αA* with 0 < *α* < 2/*σ*_1_^2^, it holds that

(25) $$\begin{array}{c} ||A\left(V_{0}-A^{†}\right)||<1. \end{array}$$

We are about to use this fact in the following theorem so as to find the theoretical order of the proposed method (7) for finding the Moore-Penrose inverse (see [16] for more details).

**Theorem 4**

For *A* ∈ *ℂ* ^*m*×*k*^, with the singular values *σ*_1_ > *σ*_2_ > ⋯*σ*_*r*_ > 0, the sequence {*V*_*n*_}_*n*=0_^*n*=*∞*^ generated by (7) and using the initial approximation *V*_0_ = αA* converges to the Moore-Penrose inverse *A* ^†^ in twelfth order provided that 0 < *α* < 2/*σ*_1_^2^.

**Proof**

Set *𝔼*_*n*_ = *V*_*n*_ − *A* ^†^ and *E*_*n*_ = *I* − *AV*_*n*_; we have

(26) A𝔼n+1=AVn+1−AA†=AVn+1−I+I−AA†=−En+1+I−AA†=−116[27En12+27En13+9En14+En15]+I−AA†.

On the other hand, from the definitions of the Moore-Penrose inverse *A* ^†^, we have

(27) $$\begin{array}{c} {\left(I-AA^{†}\right)}^{k}=I-AA^{†},\;k=1,2,\ldots ;\\ \left(I-AA^{†}\right)A{𝔼}_{n}=0. \end{array}$$

The use of these relations implies that

(28) A𝔼n+1=116[−27(A𝔼n)12+27(A𝔼n)13 −9(A𝔼n)14+(A𝔼n)15].

So, for any matrix norm ||·||, we obtain

(29) ||A𝔼n+1||≤116[27||A𝔼n||12+27||A𝔼n||13 +9||A𝔼n||14+||A𝔼n||15].

Applying (25), which implies that ||*A𝔼*_0_|| < 1, and a similar reasoning as in (10)–(12), one can obtain

(30) ||A𝔼n+1|| ≤116[27||A𝔼n||12+27||A𝔼n||13+9||A𝔼n||14+||A𝔼n||15] ≤||A𝔼n||12≤||A||12||𝔼n||12.

Finally, using the properties of the Moore-Penrose inverse *A* ^†^ and Theorem 2, it would be now easy to find the error inequality of the new scheme (7) as follows:

(31) ||Vn+1−A†||=||A†AVn+1−A†AA†||≤||A†||||AVn+1−AA†||=||A†||||A𝔼n+1||≤||A†||||A||12||𝔼n||12.

Thus, ||*V*_*n*_ − *A* ^†^|| → 0; that is, the sequence of (7) converges to the Moore-Penrose inverse in twelfth order as *n* → +*∞*. This ends the proof.

## 3. Stability

We investigate the stability of (7) for finding *A* ^†^ (or the simplified case *A* ^−1^) in a neighborhood of the solution of equation *AV* = *I*. Note that if the iteration is not self-correcting, that is, if errors made at one stage are not subsequently damped, then the inevitable rounding errors introduced into the iteration may accumulate to the point where they overwhelm the answer. Thus, we should either show that the proposed method is self-correcting or must furnish an analysis showing that rounding errors remain under control. This will be done in what follows. In fact, we analyze how a small perturbation at the *n*th iterate is amplified or damped along the iterates. Note that this procedure has recently been applied on a general family of methods for matrix inversion in [17].

**Theorem 5**

The sequence {*V*_*n*_}_*n*=0_^*n*=*∞*^ generated by (7) with the initial approximation (19) is asymptotically stable for finding the Moore-Penrose generalized inverse.

**Proof**

Let Δ*V*_*n*_ be the numerical perturbation introduced at the *n*th iterate of (7). Next, one has

(32) $$\begin{array}{c} {\tilde{V}}_{n}=V_{n}+\Delta V_{n}. \end{array}$$

Here, we perform a first-order error analysis; that is, we formally neglect quadratic or higher terms such as (Δ*V*_*n*_)^2^. This formal manipulation is meaningful if Δ*V*_*n*_ is *sufficiently small* and further yields to *V*_*n*_ · Δ*V*_*n*_ ≈ Δ*V*_*n*_ · *V*_*n*_. We have

(33) V~n+1=164V~nϕ(V~n)=164V~n(816I−4812AV~n+17393(AV~n)2−43044(AV~n)3 +77154(AV~n)4−103312(AV~n)5+105039(AV~n)6 −81576(AV~n)7+48268(AV~n)8−21516(AV~n)9 +7071(AV~n)10−1652(AV~n)11+258(AV~n)12 −24(AV~n)13+(AV~n)14)=164(Vn+ΔVn) ×(816I−4812A(Vn+ΔVn)+17393(A(Vn+ΔVn))2 −43044(A(Vn+ΔVn))3+77154(A(Vn+ΔVn))4 −103312(A(Vn+ΔVn))5+105039(A(Vn+ΔVn))6 −81576(A(Vn+ΔVn))7+48268(A(Vn+ΔVn))8 −21516(A(Vn+ΔVn))9+7071(A(Vn+ΔVn))10 −1652(A(Vn+ΔVn))11+258(A(Vn+ΔVn))12 −24(A(Vn+ΔVn))13+(A(Vn+ΔVn))14)≈164(Vn+ΔVn) ×(816I−4812(AVn+AΔVn) +17393((AVn)2+2A(AVn)1ΔVn) −43044((AVn)3+3A(AVn)2ΔVn) +77154((AVn)4+4A(AVn)3ΔVn) −103312((AVn)5+5A(AVn)4ΔVn) +105039((AVn)6+6A(AVn)5ΔVn) −81576((AVn)7+7A(AVn)6ΔVn) +48268((AVn)8+8A(AVn)7ΔVn) −21516((AVn)9+9A(AVn)8ΔVn) +7071((AVn)10+10A(AVn)9ΔVn) −1652((AVn)11+11A(AVn)10ΔVn) +258((AVn)12+12A(AVn)11ΔVn) −24((AVn)13+13A(AVn)12ΔVn) +((AVn)14+14A(AVn)13ΔVn))≈Vn+1+514Vn−120316(AVn)1Vn+1739364(AVn)2Vn −1076116(AVn)3Vn+3857732(AVn)4Vn −64574(AVn)5Vn+10503964(AVn)6Vn−101978(AVn)7Vn +1206716(AVn)8Vn−537916(AVn)9Vn +707164(AVn)10Vn−41316(AVn)11Vn+12932(AVn)12Vn −38(AVn)13Vn+164(AVn)14Vn+514ΔVn−12038AVnΔVn +5217964(AVn)2ΔVn−107614(AVn)3ΔVn +19288532(AVn)4ΔVn−193712(AVn)5ΔVn +73527364(AVn)6ΔVn−10197(AVn)7ΔVn +10860316(AVn)8ΔVn−268958(AVn)9ΔVn +7778164(AVn)10ΔVn−12394(AVn)11ΔVn +167732(AVn)12ΔVn−214(AVn)13ΔVn +1564(AVn)14ΔVn,

where (Δ*V*_*n*_)^*i*^ ≈ 0, *i* ≥ 2 has been used, since they are very close to the zero (matrix). After some algebraic manipulation and using $\Delta V_{n+1}\approx {\tilde{V}}_{n+1}-V_{n+1}$, we have

(34) ΔVn+1 ≈364(4I−AVn)2(I−AVn)11(17I−5AVn)ΔVn ≈364[(3I+I−AVn)2(I−AVn)11(12I+5(I−AVn))]ΔVn, ≈364[108(I−AA†)11+117(I−AA†)12     +42(I−AA†)13+5(I−AA†)14]ΔVn,

by using the fact that for enough large *n*, we have *V*_*n*_ ≈ *A* ^†^. We attain

(35) ||ΔVn+1||≤364[108||I−AA†||11+117||I−AA†||12  +42||I−AA†||13+5||I−AA†||14]||ΔVn||.

From (35), we can conclude that the perturbation at the iterate *n* + 1, is bounded. Therefore, the sequence {*V*_*n*_}_*n*=0_^*n*=*∞*^ generated by (7) is asymptotically stable. This ends the proof.

**Corollary 6**

By using the matrix identity in (27), it would be possible to further simplify bound (35) as follows:

(36) $$\begin{array}{c} ||\Delta V_{n+1}||\leq \left(\frac{51}{4}||I-AA^{†}||\right)||\Delta V_{n}||. \end{array}$$

Using (36) recursively, one may attain the following very simple bound:

(37) $$\begin{array}{c} ||\Delta V_{n+1}||\leq \left[{\left(\frac{51}{4}\right)}^{n+1}||I-AA^{†}||\right]||\Delta V_{0}||. \end{array}$$

**Remark 7**

In case of finding the regular inverse of nonsingular matrices, that is, when *A* ^†^ = *A* ^−1^, according to (37), we have Δ*V*_*n*+1_ ≈ 0, and so the matrix method is strongly numerically stable. Consequently, in case of finding the *A* ^†^, the matrix method (7), is asymptotically stable. Of course, since the iteration is not self-correcting in the general case, proceeding beyond convergence may cause a serious increase in error.

## 4. Initial Value

The iterative methods that have been discussed up to now are sensitive upon choosing the initial guess/value to start the process. As a matter of fact, the high accuracy and efficiency of such types of iterative algorithms are guaranteed only if the initial value satisfies the appropriate condition given in Theorem 1. Thus, in order to preserve the convergence order, we present some ways from the literature to remedy this flaw, although an efficient way for square or rectangular matrices is the way (19).

For a symmetric positive definite (SPD) matrix *A*, one can easily use the Householder-John Theorem to attain

(38) $$\begin{array}{c} V_{0}=P^{-1} \end{array}$$

as the initial value, wherein the matrix *P* is any of the matrices such that *P* + *P* ^*T*^ − *A* is SPD [8].

If the square matrix *A* is diagonally dominant, one may apply the approach given in [18] and use

(39) $$\begin{array}{c} V_{0}=\mathrm{diag}\left(\frac{1}{a_{11}},\frac{1}{a_{22}},\ldots ,\frac{1}{a_{nn}}\right), \end{array}$$

wherein *a*_*ii*_ is the diagonal entry of *A*. Note that this choice is so much fruitful in solving PDEs resulting from discretizations. Some further generalizations of such an initial matrix are given in [19].

Although the two abovementioned ways are efficient, they cannot be applied for finding an initial guess/value to the inverse of general input matrices. For instance, for large-scale matrices which do not satisfy the above structures, they may fail to provide the convergence. Hence, we here take into account the suboptimal way of producing *V*_0_ as given by Pan and Schreiber in [20] as follows:

(40) $$\begin{array}{c} V_{0}=\frac{A^{T}}{{||A||}_{1}{||A||}_{\infty }}. \end{array}$$

We should note that choosing the initial value as given above can satisfy the necessary condition of arriving to the convergence phase. Some ways for updating the initial matrix for sparse matrices are brought forward by [21].

In what follows, we provide an algorithm for improving an initial matrix for square matrices rapidly. In fact, the derivation of LU factorization for almost all kinds of square nonsingular matrices could be done rapidly in the linear algebra programming packages. In the Mathematica, the one argument command LinearSolve[ ] provides an LU factorization of *A* too much fast. Then, by applying the LU decomposition on the columns of a identity matrix recursively, one could update the columns of a derived initial matrix produced by other strategies such as (40). Such a procedure is illustrated in Algorithm 1. We summarized this idea as in Algorithm 2.

The initial1[A_,num_] takes the nonsingular matrix *A* and the number of columns that users wish to update from the real inverse into the approximate inverse from the left, while the function initial2[A_,num_] works doubly. That is, if, for example, num=10, it updates the first and last 10 columns of the approximate inverse as rapidly as possible.

Next, we conduct some numerical tests to support the theoretical results given in Section 2 using the initial value discussed herein.

## 5. Computational Aspects

In this section, some experiments are presented to demonstrate the capability of the proposed method. The programming package mathematica 8 [22] has been used in the demonstrations. We work on the numerical aspects of the methods in machine precision.

It is clear that large sparse matrices cannot be handled easily and their storage needs to be done in sparse form as in the input form to be accessible and economic in real applications. Methods like (7) are powerful in finding an approximate inverse or a robust approximate inverse preconditioner in low number of steps and computational time, in which the output form of the approximate inverse is also * sparse*.

In this paper, as the programs were running, we found the running time using the command AbsoluteTiming[ ] to report the elapsed CPU time (in seconds) for this experiment. In this paper, the computer specifications are Microsoft Windows XP Intel(R), Pentium(R) 4, and CPU 3.20 GHz, with 4 GB of RAM.

**Experiment 1**

This test is devoted to the application of the Schulz-type iterative methods in finding the pseudoinverse of 30 large random complex matrices defined as shown in Algorithm 3 ($I=\sqrt{-1}$).

**Figure 1:**
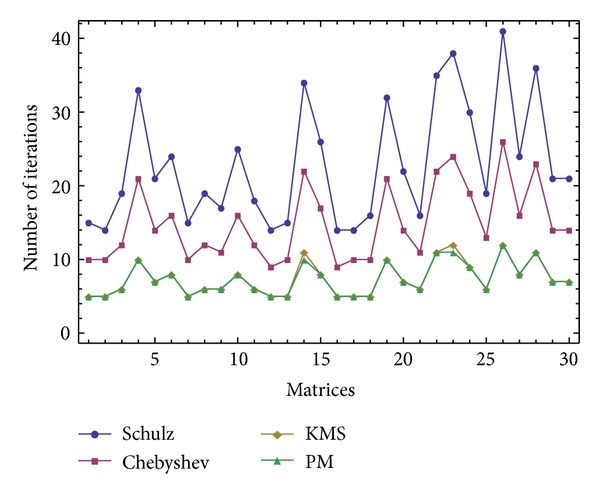
The results of comparisons for Experiment 1 in terms of the number of iterations.

**Figure 2:**
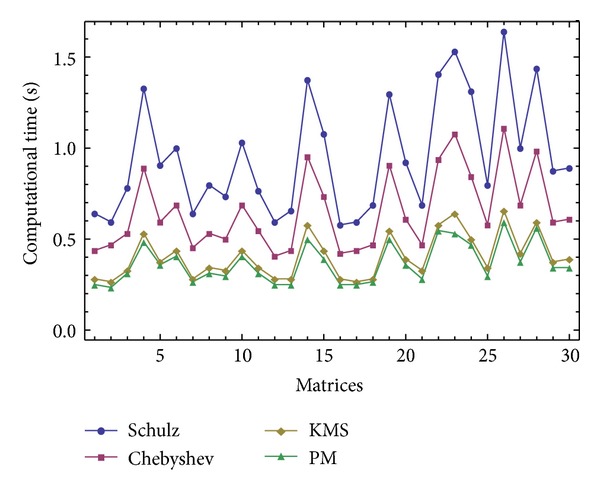
The results of comparisons for Experiment 1 in terms of the elapsed time using ||*V*_*n*+1_−*V*_*n*_||_*∞*_ ≤ 10^−6^.

**Figure 3:**
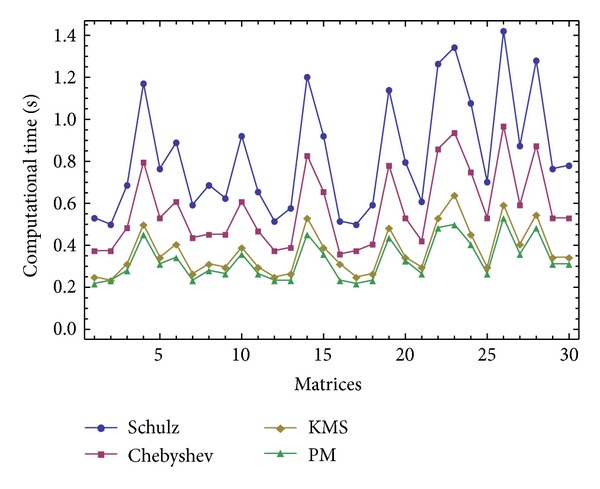
The results of comparisons for Experiment 1 in terms of the elapsed time using ||*V*_*n*+1_−*V*_*n*_||_*F*_ ≤ 10^−6^.

The results of comparisons for these random matrices of the size *m* × *k* = 1500 × 1800 are reported in Figures 1, 2, and 3 in terms of the number of iterations and the computational time. The compared methods are (1) denoted by “Schulz,” (2) denoted by “Chebyshev,” (4) denoted by “KMS,” and the new iterative scheme (7) denoted by “PM." A saving in the elapsed time by considering the stopping criteria as ||*V*_*n*+1_−*V*_*n*_||_*∞*_ ≤ 10^−6^ and ||*V*_*n*+1_−*V*_*n*_||_*F*_ ≤ 10^−6^ can be observed for the studied method (7). In this test, the initial matrix has been computed for each random matrix by V_0_[j]=ConjugateTranspose[A[j]] ∗(1./((SingularValueList[A[j],1][[1]])^2^)), while the maximum number of iterations is set to 100.

## 6. Concluding Remarks

It is well known that matrix inverse and generalized inverse are important in applied fields of nature science, such as the solution to various systems of linear and nonlinear equations, eigenvalue problems, and the linear least square problems. Iterative methods are often effective especially for large-scale systems with sparsity and Schulz-type methods are great tools for preconditioning such systems or in finding pseudoinverses.

Hotelling-Bodewig algorithm is simple to describe and analyze and is numerically stable. This was the idea of developing an iterative method of this type in this paper.

In this paper, we have shown that the suggested method (7) reaches twelfth order of convergence. The stability of the new method was also studied in detail and established that the new scheme is asymptotically stable. The efficacy of the new scheme was illustrated numerically in Section 5. Finally, and based on the numerical results obtained, one can conclude that the presented method is useful. Further extensions of the new scheme for other generalized inverses (such as the ones in [23, 24]) can be done for future works.

## Figures and Tables

**Algorithm 1:**
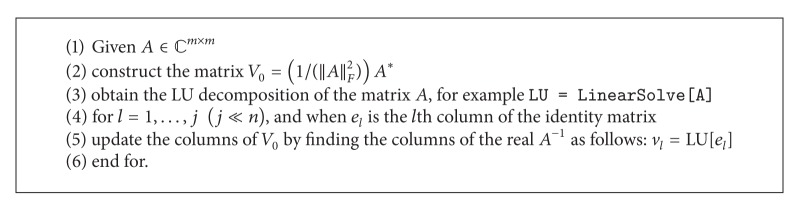
An algorithm for constructing a rapid and robust initial approximation for *A* ^−1^.

**Algorithm 2:**
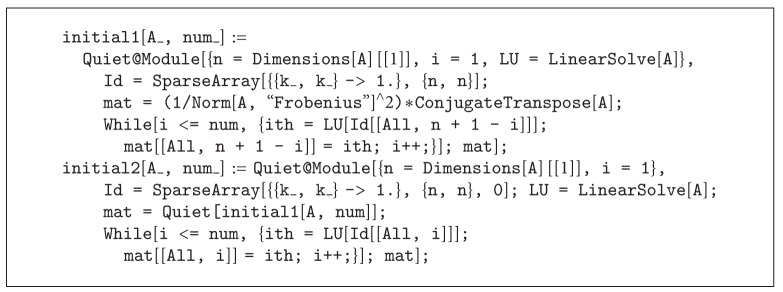
Two-argument function written in the Mathematica environment.

**Algorithm 3:**
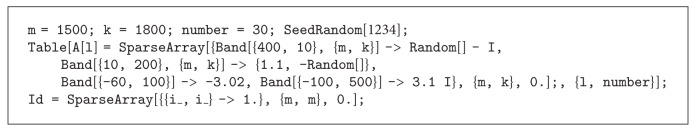
titleworktilte
